# Albuminuria as Bearing on Puerperal Eclampsia

**Published:** 1897-10

**Authors:** E. V. Ross

**Affiliations:** Rochester, N. Y.


### ALBUMINURIA AS BEARING ON PUERPERAL ECLAMPSIA.

E. V. Ross, M. D., Rochester, N. Y.

A case of albuminuria, complicating pregnancy, and terminating in death, came to my notice quite recently, and the lesson that it taught is my only excuse for this short paper.

Mrs. M. F., æt. thirty-five, in her second pregnancy. About four weeks prior to her expected confinement she consulted her physician with the statement of not feeling well. An examination of the urine was made, and a large amount of albumen discovered.

She was placed upon a milk diet, and other treatment instituted. A week later only a small amount was detected, and it continued to disappear from the urine. Ten days after date of confinement the urine was again examined for the last time, and no albumen found. A favorable termination was therefore looked for, but two days later the patient complained of objects appearing as in a mist, or as if she was looking through a veil (uremia amblyopia). This symptom came on quite suddenly, and gradually increased; ten hours later she became comatose and died, evidently from uremic poisoning.

There are other cases on record that tend to show that we must not place too much reliance upon the disappearance of albumen from the urine as a favorable sign. Most writers are agreed at the present that puerperal eclampsia is due to some toxic or effete material retained in the system; just what this is there is a difference of opinion. Parvin, in the last (third) edition of his work, has this to say: "What this poison is, or conceding more than one toxic agent, what these poisons are, and what the origin, are questions still unanswered."* He further says:† "The nervous theory, that which made the disease the result of cerebro-spinal congestion, the uremic theory and its derivatives—have passed away. * * * The theory which marks the essential cause toxemia—not one, but several different poisons, it may be, concerned—is now generally upheld. So, too, the toxemia, while usually associated with renal failure, and dependent upon it, does not in all cases have such association and dependence, for the disease caused by the toxemia may occur without renal disorder; moreover, it is a question in some cases whether this disorder is not the consequence rather than the cause of the toxic condition."

Whether the toxic agent, or agents, be the cause or result of the kidney lesion one thing is quite evident, viz., that the presence or absence of albumen from the urine is of no paramount importance as indicating the extent of renal insufficiency whereby these toxic products (one or more?) are retained. In further proof of the statement we bring forth the following as evidence:

(1) That albuminuria occurs in 5 to 10 per cent. of all pregnant women.

(2) That puerperal eclampsia occurs once in about 260 cases‡ (Vinay§).

(3) That puerperal eclampsia occurs without the presence of albumen in the urine.

(4) That albumen in the urine is not always followed by eclampsia.

(5) That in a large percentage of cases of puerperal eclampsia albumen is found only after the appearance of the convulsions.

If, then, the presence or absence of albumen in the urine

---

* *Science and Art of Obstetrics*, page 412.

† *American Text-Book of Obstetrics*, page 632.

‡ Anvard, three in 1,000; Martin and Kaltenboch, one in 500.

§ *Traité des Maladies de la Grossesse, etc.*, Paris, 1894.

of a pregnant woman is not to be taken as an index to her condition what, then, are we to judge from? In spite of what has been quoted in opposition to the uremic theory we would suggest that the urine be examined for the daily amount of urea excreted for some time before confinement in addition to the test for albumen. If physicians will take the trouble (and duty demands it) to make such examination in every case, we will soon have some definite knowledge that will have a great bearing on this vital subject.

